# Ocular Manifestations in Children with Vernal Keratoconjunctivitis

**DOI:** 10.3390/children12050609

**Published:** 2025-05-07

**Authors:** Matteo Capobianco, Marco Zeppieri, Giuseppe Gagliano, Federico Visalli, Fabiana D’Esposito, Roberta Foti, Ludovica Cannizzaro, Daniele Tognetto, Caterina Gagliano

**Affiliations:** 1 Department of Ophthalmology, University of Catania, 95123 Catania, Italy; capobiancoteo@gmail.com (M.C.); giuseppe.gagliano99@gmail.com (G.G.);; 2 Department of Ophthalmology, University Hospital of Udine, 33100 Udine, Italy; 3 Department of Medicine, Surgery and Health Sciences, University of Trieste, 34127 Trieste, Italy; 4 Imperial College Ophthalmic Research Group [ICORG] Unit, Imperial College, London NW1 5QH, UK; 5 Eye Clinic, Department of Neurosciences, Reproductive Sciences and Dentistry, University of Naples Federico II, 80131 Naples, Italy; 6 Rheumatology Unit, Policlinico San Marco Hospital, 95121 Catania, Italy; 7 Department of Medicine and Surgery, University of Enna “Kore”, Piazza dell’Università, 94100 Enna, Italy; caterina.gagliano@unikore.it; 8 Mediterranean Foundation “G.B. Morgagni”, 95125 Catania, Italy

**Keywords:** meibomian gland dysfunction, lipid layer instability, evaporative dry eye, vitamin A therapy

## Abstract

Background: Vernal keratoconjunctivitis (VKC) is a chronic, recurrent, and frequently severe allergic ocular condition predominantly impacting children and adolescents in tropical and subtropical areas. It profoundly affects patients’ quality of life owing to its chronic symptoms and possible vision-threatening effects. Notwithstanding progress in comprehending VKC, its ocular symptoms and therapeutic approaches necessitate ongoing assessment. Aims: This review summarizes the main factors to consider when diagnosing, treating, and managing patients with VKC based on the current literature in this field. Methods: This comprehensive review examined peer-reviewed literature from 2010 to 2024 obtained from PubMed. The selection criteria encompassed research addressing the clinical presentation, diagnostic difficulties, and therapy of visual symptoms in pediatric patients with VKC. The publications chosen were those focusing on those that elucidate the pathophysiology, consequences, and innovations in treatment methodologies. Results: The ocular manifestations of VKC are varied and characterized by prominent symptoms such as severe itching, photophobia, lacrimation, and a viscous mucoid discharge. Clinical manifestations range from conjunctival hyperemia and limbal thickening to severe consequences that jeopardize vision, including shield ulcers and keratoconus. Improvements in imaging techniques such as anterior segment optical coherence tomography (AS-OCT) and in vivo confocal microscopy have enhanced diagnostic accuracy. The pharmacological approach has transitioned to steroid-sparing techniques, prioritizing mast cell stabilizers, antihistamines, and immunomodulators such as cyclosporine. Novel therapies, including biologics that target interleukin pathways, demonstrate potential in refractory instances. Nonetheless, access to modern medicines is restricted in resource-limited environments. Conclusions: VKC poses considerable diagnostic and treatment difficulties due to its chronic nature and possible consequences. This review emphasizes the necessity for prompt diagnosis and customized management approaches to avert vision impairment. Despite considerable advancements in comprehending VKC’s etiology and therapy, inequalities in access to sophisticated care highlight the necessity for global activities to guarantee equitable treatment alternatives.

## 1. Introduction

Vernal keratoconjunctivitis (VKC) is a persistent, recurrent allergic ocular condition predominantly impacting infants and adolescents in tropical and subtropical areas. It results in considerable ocular morbidity and affects quality of life. Notwithstanding progress, the diagnosis and treatment of VKC continue to pose difficulties. This study aims to deliver a thorough yet concentrated summary of existing knowledge regarding VKC’s clinical characteristics, diagnostic methods, and treatment strategies to aid doctors in prompt and effective patient management [1].

### 1.1. Epidemiology and Risk Factors

The M:F ratio is 4:1 with a clinical presentation that generally occurs between 5 and 15 years of age, although cases have also been described in younger children. Spontaneous remission frequently occurs in adulthood, although in some cases it can persist beyond adolescence. The main risk factors would appear to be related to the simultaneous presence of atopy and atopic dermatitis, asthma, eczema, and allergic rhinitis. Furthermore, most patients have a positive family history of allergic diseases. Studies also show that pollution and environmental factors play a key role in VKC. In particular, NO_2_, O_3_, PM 10 and 2.5, temperature, and solar radiation, probably through mechanisms related to UV radiation and vitamin D levels, also appear to be significant factors [2,3,4].

### 1.2. Pathogenesis

VKC appears to occur following any chronic allergic stimulus in the form of internal or external allergens. This leads to the activation and degranulation of mast cells and the hyperactivity of Th2 lymphocytes. We will therefore have the release of histamine, cytokines, and proteases. The activity of Th2 lymphocytes mediates the release of IL-4 and IL-13, which are essential for the release of IgE from lymphocytes. All these cells and inflammatory mediators will be recruited to the conjunctiva and cornea, leading to chronic inflammation. Therefore, VKC is characterized by type I and type IV hypersensitivity reactions [5]. The chronic inflammatory infiltrate will subsequently lead to the activation of fibroblasts, which will proliferate and deposit extracellular matrix and fibrous cells at the level of the conjunctival stroma. All this together with the deposition of collagen and other proteins will lead to a process of hyalinization and conjunctival thickening [6,7]. During this process, the role played by some cellular mediators such as TGF beta, VEGF, and PDGF is also important. These changes will lead to an anatomical–functional modification of the bulbar and tarsal conjunctiva with the final formation of papillae. The papillae are characterized by a central vascular nucleus supported by inflammatory cells and an overlying hyperplastic and hypertrophic epithelium. Papillae in VKC are commonly seen in the upper tarsal conjunctiva. The main differential diagnosis is with AKC or atopic Keratoconjunctivitis and with allergic conjunctivitis. Compared to the latter, VKC is characterized by a more marked inflammatory component [8,9,10,11].

### 1.3. Therapy

The therapy of VKC is particularly complex as it is not easy to manage the drugs used in an acute phase and chronic phase, and at the same time be able to adopt steroid-sparing strategies. The current literature highlights, in addition to the drugs already used such as topical antihistamines, corticosteroids, and topical immunomodulators (cyclosporin A, tacrolimus), a growing interest in drugs that can selectively modulate the inflammatory process involving inflammatory mediators such as interleukin-4 (IL-4), interleukin-5 (IL-5), and interleukin-13 (IL-13). Preliminary studies have also been carried out for the use of dupilumab (anti-IL-4Rα) or mepolizumab (anti-IL-5) [12,13,14].

### 1.4. Diagnostic Advances

The steady drip of new technology has radically sharpened how we diagnose and keep tabs on VKC. Today’s front-of-the-eye imaging tools reveal the cornea and conjunctiva in striking detail, turning what once relied on gut feeling into a far more objective call and making it much easier to tell VKC from other chronic forms of conjunctivitis [3,12].

### 1.5. Inequalities in Access to Care

Despite significant therapeutic and diagnostic progress, inequalities in access to care persist. In many developing countries, the main treatment is still based on the use of topical corticosteroids, which are effective in managing the acute phase but problematic in the long term due to possible side effects. The high cost of topical immunomodulatory agents and biological therapies limits their global diffusion; therefore, global development strategies will be needed to ensure equal access to therapies in the future [15]. VKC is certainly a complex and debilitating disease, with significant implications for the quality of life of pediatric patients. To date, despite the greater understanding of the pathophysiological mechanisms and new diagnostic–therapeutic approaches, important gaps remain regarding the management of the disease, access to care, and prevention strategies for ocular and visual complications. The aim of this narrative review was to delineate and encapsulate the principal visual signs, diagnostic innovations, and therapeutic approaches in VKC, which can be of clinical use when managing these patients in a routine clinical setting.

## 2. Materials and Methods

This study is a comprehensive literature review, conducted to investigate the main ocular manifestations, diagnostic challenges, and main therapies in pediatric patients with VKC. The literature search strategy aimed to optimize sensitivity by identifying all potentially relevant articles and to improve specificity by implementing stringent inclusion criteria exclusively targeting pediatric VKC studies related to ocular manifestations, diagnostic tools, and therapies. We searched through PubMed for papers on vernal keratoconjunctivitis (VKC) in children, covering January 2010 to February 2024. Using search phrases like “vernal keratoconjunctivitis” and “pediatric ocular allergy”, we kept only English-language studies on patients 18 or younger. We included original research, reviews, guidelines, and solid case series but excluded adult-only work, non-English pieces, abstracts, opinion articles without data, and letters. The first search included 152 records; after browsing the titles and abstracts, we kept 74 records. A deeper read narrowed this down further to 62 studies that matched all the criteria. Two reviewers processed the screening on their own, talked through any disagreements, and removed duplicates by hand. PubMed was our source because of its broad biomedical reach, though we admit that searching Embase or Scopus might have provided more insightful works. The included studies met the following precise criteria: concerned pediatric patients; examined ocular manifestations from a clinical, diagnostic, and therapeutic point of view; analyzed diagnostic methods and technological advances in detail, such as the use of confocal microscopy and AS-OCT; and evaluated pharmacological interventions and possible new therapies, focusing on steroid-sparing strategies.

The data collected were extracted and used independently by experienced researchers using a standardized form to ensure uniformity in data collection. Any discrepancies found were resolved through discussion and the guidance of other reviewers. As this review was based on already published data, the ethics committee was not required. Nevertheless, this review was conducted with the utmost respect for current regulations and the ethical standards of the scientific community. In summary, our review, characterized by maximum transparency in the selection and analysis of scientific data and replicability, aims to provide a complete and comprehensive overview of vernal keratoconjunctivitis and future diagnostic–therapeutic challenges.

## 3. Results

Below, we will report and discuss the results that emerged from the analysis of the currently available literature, with a critical analysis of the available data. Considering the great variety and variability of the data collected, the section will be organized into sub-sections in such a way as to facilitate its usability and give greater order to the analysis performed.

## 4. Clinical Manifestations and Management of Vernal Keratoconjunctivitis (VKC)

### 4.1. Clinical Manifestations

Because it is a complex disease, VKC has great phenotypic variability. Clinical pictures range from simple ocular discomfort to severe alterations of the anterior surface. The most common symptoms include:

- Conjunctival hyperemia and chemosis, often the first ocular manifestations.
- Ocular itching, often intense, persistent, and refractory to topical antihistamine therapy.
- Papillae, preferentially in the superior tarsal conjunctiva.
- Foreign body sensation, often due to the presence of papillae.
- Heaviness of eyelids.
- Photophobia.
- Abundant tearing and mucous secretion, typically white, thick, and stringy.
- Thickening of the limbus, with papillae and Horner–Trantas nodules.
- Ptosis and blepharospasm, when the papillae become so large and heavy that they interfere with the normal functionality of the anatomical structures.
- Pain and diminution of visual acuity only if corneal ulcers are present.
- Chronic and recurrent bilateral conjunctivitis with conjunctival hyperemia and alterations of the corneal limbus.

Seasonal trend with worsening of symptoms in the warm months; however, this is not a rule since there are cases of perennial VKC [16,17].

Considering the clinical and histopathological characteristics, VKC can be divided into three main forms.

#### 4.1.1. Palpebral Form

This form is usually more common in the European and US population, characterized by the presence of multiple, confluent, giant papillae usually on the upper tarsal conjunctiva. These papillae, of variable size but usually large, give a “cobble stone or pavement stone fashion” appearance due to the presence of hard flat-topped surface. A stringy, whitish secretion can often be observed between the papillae, often associated with eyelid ptosis and secondary corneal abrasions.

Papillae can be classified according to their size:

○ Grade 1: from 0.1 mm to 1 mm.
○ Grade 2: from 1 mm to 3 mm (micropapillae).
○ Grade 3: >3 mm (giant papillae).

Another characteristic element of the palpebral shape is the deposition of fibrin between the papillae, therefore resulting in the formation of pseudo-membranes (Maxwell-Lyons sign). In palpebral forms, it is always advisable to use a dye such as fluorescein during the objective examination. This is particularly useful for two reasons: first, especially for grade 1 forms, it makes the papillae more visible, and second, epithelial stippling indicates disease activity. In this regard, there are various classifications for evaluating the pattern given by staining, such as the Oxford classification, Van Bljsterweld, and the KC-Collaborative Longitudinal Evaluation of Keratoconus study (CLEK) (VKC-CLEK) scores [18,19,20].

#### 4.1.2. Limbic/Bulbar Form

This form is characterized by the presence of nodules at the level of the limbus, which are nothing more than particular papillae with a gelatinous–mucous appearance. When several papillae merge together, it is possible to observe a gelatinous thickening of the lima. Another characteristic of this form is the presence of Horner–Trantas nodules, more frequent in subjects of African and Asian origin, which we find above or next to the papillae. These small, white, chalky nodules are important because they indicate that the disease is active and serious. In fact, these nodules are composed of degenerated epithelial cells and eosinophils, which are particularly harmful to the cornea. Eosinophils are in fact able to produce epithelial–toxic enzymes such as eosinophil cationic protein, eosinophil peroxidase, myelin basic proteins, and eosinophil granule proteins. These enzymes can damage the corneal surface at the limbus and lead to long-term complications such as neovascularization and corneal pannus. A dark red triangular congestion of the bulbar conjunctiva in the palpebral area may also be present.

#### 4.1.3. Mixed Form

More common in tropical countries and India, this form combines the clinical signs of the palpebral and limbal forms. It is characterized by a high risk of corneal complications [21,22].

Therefore, depending on the anatomical structures involved, we will have a form with conjunctival involvement, a form with limbal involvement, and a mixed form. Three aspects must also be considered.

##### Corneal Involvement

*Damage* to the cornea increases the severity of the pathology and increases the possibility of complications. Corneal changes are variable and range from punctate epithelial keratitis (PEK) to epithelial erosions to corneal ulcer formation, typically with a shield-like appearance, as well as sub-epithelial/stromal scarring. Corneal ulcers must always be treated appropriately to avoid superinfections, corneal scarring, and decreased visual acuity. Corneal ulcers can be classified clinically according to different classifications. There are six different grades, grade A and grade B classify the epithelial erosions while grade C or grade 1, grades D and E or grade 2, and grade F or grade 3 classify the epithelial defect. Rarely, next to the ulcer, due to the presence of chronic inflammation, deposits consisting of mucus and inflammatory debris will form, which can lead to the formation of a plaque. This must be treated with a need for debridement as it prevents the correct re-epithelialization and, therefore, the healing of the ulcer. Finally, another corneal manifestation associated with VKC is represented by pseudogerontoxon. It is a stromal deposition in the peripheral corneal portion of grayish appearance secondary to the inflammatory process. In fact, while gerontoxon is related to the deposition of lipids, pseudogerontoxon is related to the deposition of inflammatory cells [23,24].

##### Eyelid Skin Involvement

This is important because VKC rarely involves the skin, in which case it is necessary to pay close attention and make a differential diagnosis with atopic keratoconjunctivitis or AKC [25].

##### Peri-Ocular Pigmentation

**Figure 1:**
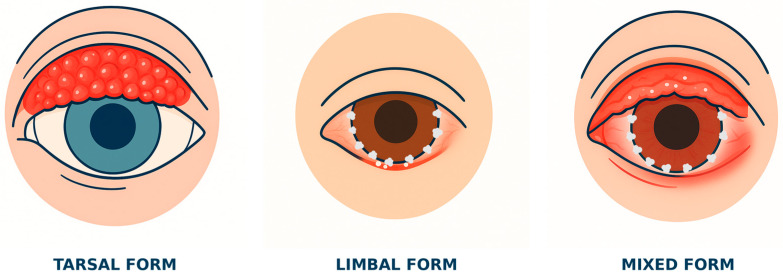
Schematic representation of the typical clinical manifestations of vernal keratoconjunctivitis (VKC) in the tarsal, limbal, and mixed forms, which include giant papillae usually on the upper tarsal conjunctiva, nodules at the level of the limbus, and congestion of the bulbar conjunctiva.

Pigmentation at the level of the bulbar or tarsal conjunctiva is an important sign of chronic inflammation. This hyperpigmentation, also called allergic shiner or infra-palpebral hyperpigmentation, would seem to be related to the constant rubbing of the papillae and the infiltration of inflammatory mediators. Inflammation is also responsible for venous stasis and stimulation of melanogenesis. These two elements favor a “dark bluish” appearance in the affected area and the deposition of hemosiderin [23,26]. The typical clinical manifestations are shown in Figure 1.

### 4.2. Classification

To date, there is no univocal way to classify VKC, and the morphological classification is still valid. However, over the years, several authors have tried to offer a classification based on clinical severity in order to stratify the pathology more precisely and therefore use personalized and targeted therapies based on severity. One of the most recent classifications proposed by Zicari et al., based in part on the “historical” classification of Bonini et al., divides VKC into four or five levels of severity (depending on the version and clinical adaptation) [27]. Table 1 shows the classification of VKC.

### 4.3. Complications and Consequences for Vision

If not adequately treated, VKC can lead to serious complications of the ocular surface with potential and irreversible consequences for vision [28,29,30,31,32,33]. The main complications include:

- Persistent epithelial defect.
- Giant corneal ulcers (shield ulcers).
- Keratoconus: secondary to the chronic inflammatory state and continuous ocular rubbing.
- Corneal neovascularization and scarring: in advanced cases, the alterations can lead to a permanent reduction in visual acuity.
- Corneal pannus.
- Corneal opacity and scarring: recurrent inflammatory and ulcerative processes can lead to the formation of corneal leukomas and sub-epithelial/stromal scarring.
- Cataracts and glaucoma due to steroids.
- Limbal stem cell deficiency.

### 4.4. Advanced Diagnostic Methods

From the review of the literature conducted, it is clear that the use of instrumental techniques and technologies is increasingly widespread to increase our diagnostic and differential diagnosis capacity. It is certainly essential to start by visiting the patient, carefully and precisely collecting the anamnesis, and completing the visit with biomicroscopic examination with the slit lamp. The evaluation of the patient with slit lamp biomicroscopy remains a key and fundamental examination to perform. It allows for the direct evaluation of the ocular surface and anatomical structures and the detection of papillae, limbal thickening, and Trantas nodules. However, it is clear that thanks to the help of these technologies, we are able to identify microscopic and subclinical alterations that precede macroscopically evident clinical alterations, allowing us to diagnose and treat the patient early. Furthermore, the help and use of new methods with a multimodal imaging approach allow us to have a quantity of data that helps the clinician classify the severity of the pathology with greater objectivity. Scales that are based on a subjective assessment, such as the one proposed by Zicari et al., still have their usefulness in the diagnostic–therapeutic process but are subject to excessive variability given by the subjective element of the different observers, something that is instead overcome by new technologies [28,34]. The diagnostic tools used in VKC are summarized in Table 2.

#### 4.4.1. Assessment of the Tear Film and the Ocular Surface

VKC, like other allergic diseases, leads to a compromise of the tear film. Since it is a pediatric population, although traditional tests such as the Schirmer test, tear film breakup time (TBUT), and measurement of tear osmolarity are useful, in these cases, it is wise to use machines capable of measuring the tear film breakup time in a non-invasive manner (NI-BUT) [35]. The various devices on the market are able to measure the stability of the tear film in a more precise and non-invasive manner, often not requiring the use of dyes such as fluorescein. Furthermore, by using optical devices that analyze the tear film reflex without introducing foreign substances into the eye, NI-BUT is a method that respects normal ocular physiology more and also allows for better visualization and analysis of the meibomian glands and therefore any meibomian gland dysfunction (MGD) [36]. Recently, another method based on tear film interferometry has been gaining acceptance for the possibility of performing a precise assessment of tear dynamics and evaporation rate. However, since it is a pediatric population, it is not always easy to implement these technologies due to the lack of collaboration and the not always rapid analysis times [37]. Therefore, the traditional TBUT assessment is still widely used today due to its greater ease of use and because many centers do not have other equipment [38].

#### 4.4.2. Anterior Segment Optical Coherence Tomography (AS-OCT)

It is based on the same principles as traditional OCT. In this case, longer wavelengths are used to analyze the ocular surface and cornea with high resolution. Furthermore, since it is a method that does not require any contact, it is particularly useful for a pediatric population. In VKC, AS-OCT is particularly useful for:

Detecting any irregularities of the corneal epithelium, edema and other anomalies.

Highlighting initial thinning and sub-epithelial/stromal scarring, signs that could be used to predict and anticipate the possible development of shield ulcers or keratoconus. In particular, for the latter, some studies suggest that AS-OCT is able to visualize early, even before corneal topography, any Vogt striae, and limbal hypertrophy.

Monitoring the patient with corneal complications such as ulcers over time. Furthermore, being able to perform measurements on the acquired scans, it is possible to perform follow-ups on qualitative and quantitative data.

The high availability of OCT methods and the relatively rapid learning curve to best use this machine make AS-OCT an excellent method to study patients affected by VKC [39,40]. In vivo confocal microscopy: In vivo confocal microscopy (IVCM) gives clinicians a painless, non-invasive window into the eye. By generating “virtual histology” images, it shows corneal and conjunctival cells in crisp, high-resolution detail and neatly rounds out the information obtained with AS-OCT. In patients with vernal keratoconjunctivitis, IVCM usually discloses a brighter-than-normal corneal epithelium along with a clear rise in dendritic cells. In VKC, IVCM highlights in most cases corneal epithelial hyper-reflectivity and an increase in dendritic cells. IVCM can be particularly useful for:

Monitoring whether there is a therapeutic response by analyzing the number of inflammatory cells before and after treatment.

Analyzing inflammatory cells and infiltrates.

Analyzing the corneal nerve plexus and its alterations.

Analyzing the cornea even when the latter is particularly compromised and not well visualized with other methods.

Evaluating corneal signs that can anticipate the appearance of clinically evident keratopathies.

It is also a repeatable test that does not require any contact. However, it requires the collaboration of the patient and a staff expert in its use, and not all centers have this technology. The future development of possible portable devices and machines with better-performing image acquisitions could lead IVCM to become an increasingly crucial method in the diagnosis and follow-up of VKC [41,42].

#### 4.4.3. Corneal Topography and Tomography

This technology is particularly useful in VKC, especially for the management of complications such as keratoconus and shield ulcers [43,44,45].

In the case of cherectasis and keratoconus, it is an essential test to evaluate the corneal thickness, the irregularities of the anterior and posterior corneal curvature, and the corneal asymmetry. Early recognition of this complication in pediatric patients affected by VKC allows for timely intervention. Furthermore, thanks to the quantitative data that the test provides us, such as asymmetry indices, the Inferior–Superior [I-S] ratio, and elevation maps, it is possible to monitor the patient and the therapeutic response.

In cases of ulcers and/or shield ulcers or peripheral corneal thinning, the examination is able to highlight corneal changes both when the pathology is clinically evident but also in the sub-clinical phases.

#### 4.4.4. Laboratory Investigations and Biomarkers

Although the correlation between VKC and systemic allergic sensitization is not always linear, some studies have used skin prick tests, total and splicing IgE levels, and conjunctival provocation tests (SAC, specific allergen challenge) to try to identify specific allergens potentially responsible for contributing to the exacerbation process. This can be a useful support for educating the patient and implementing strategies to reduce exposure to the antigen [46,47]. Other studies have used conjunctival scraping and cytological examination to evaluate in detail the cellular infiltrates involved and the number and density of goblet cells. It was highlighted that in the acute and active phase of the disease, there was a notable increase in mast cells and eosinophils and metaplasia of the conjunctival goblet cells. In addition, the tear film was also evaluated, highlighting high levels of IL-4, IL-5, and IL-13, inflammatory cytokines produced by Th2 lymphocytes that play a crucial role in the physiopathology of VKC [48].

Another possible role would seem to be played by IL-17-secreting T lymphocytes, especially in chronic inflammatory processes of the ocular surface. Finally, the Microarray-based IgE detection technology was also used on the tears of affected patients. Another study showed a significant decrease in the levels of CD14, TLR-4, and TLR-9 molecules on the ocular surface in the inflammatory phase [49]. Another emerging and potentially significant element in VKC could be related to the ocular microbiome [50]. Yet another study evaluated changes in the expression of those mucin-associated genes during the acute inflammatory phase and showed significant alterations in the expression of these genes. Again, as possible biomarkers to differentiate acute from chronic pathology, a simultaneous assay of CCL24/eotaxin-2, CCL17/TARC, and interleukin-16 in the tear film has been proposed [51,52]. Another possible biomarker could be Alpha-1 antitrypsin, an acute-phase protein whose levels rise dramatically during the acute phase and decrease following treatment [53]. In VKC patients, it would appear that the predominant bacterium is Staphylococcus. In addition, patients had a higher bacterial load and a higher rate of resistance to fluoroquinolones [54]. All these tests are technologically advanced and represent useful aid and support in understanding the physiopathology of the disease; however, they are not feasible on a large scale due to their invasiveness and the need for a specialized laboratory, nor in contexts with limited resources.

#### 4.4.5. Genetic and Epigenetic Markers

Some studies have evaluated some polymorphisms as possible predisposing factors for the development of VKC. Specifically, the polymorphisms concerned the genes encoding the histamine receptors and the α chain of the IL-13 and IL-4 receptors. Furthermore, the severity and course of the disease could be influenced by epigenetic modifications such as the methylation of the genes regulating Th-2 cytokines. It would also appear that during the acute inflammatory phase, there is an overexpression of genes related to protein misfolding and endoplasmic reticulum stress, pathways involved in the perpetuation of the inflammatory state. Another study evaluated changes in the expression of those mucin-associated genes during the acute inflammatory phase and showed significant alterations in the expression of these genes [55,56].

A possible gene related to the pathogenesis of VKC could be Visual System Homeobox 1 (VSX1), although further confirmatory studies are needed [57]. A possible role could also be played by microRNAs. Indeed, a preliminary study has identified some possible genes involved in the inflammatory process of VKC, namely PRELP, IL17RC, FOXP3, and SOCS3. The study also identified a number of miRNAs, of which the main ones were found to be hsa-miR-1229-5p and hsa-miR-4298, that could potentially be used as biomarkers [58]. Genetic investigations, although not part of the standard examination, represent a useful aid for the better understanding and stratification of the disease, and in the future, they may allow for the early identification of patients at risk of developing severe forms of the disease.

### 4.5. Treatment

The therapy of VKC aims to control inflammation, reduce clinical symptoms, and prevent complications. The main topical therapeutic strategies include the following [13,14,16,34,59,60]:

- Mast cell stabilizers (sodium cromoglycate 2% or 4%, lodoxamide 0.1%, N-Acetyl-aspartyl-glutamate 6%, nedocromil 2%), topical antihistamines (olopatadine, cetirizine, Levocabastine, Emedastine) dual-acting agents with a combined mechanism of mast cell stabilizers and antihistamines (olopatadine 0.1%, 0.2%, or 0.7%; bepotastine besilate 1.5%; azelastine 0.05%; alcaftadine 0.2%), ocular lubricants (without preservatives), and mucolytic agents (Acetyl Cysteine 0.5%).
- Topical NSAID (Ketorolac 0.3% or 0.5%).
- Topical steroids: Mainly used in the acute phases, to be avoided in the long term due to possible side effects such as cataracts and glaucoma. Can be divided according to potency into low-potency (Fluorometholone 0.1%, Loteprednol Etabonate 0.5%, Rimexolone 1%) and high-potency corticosteroids (Prednisolone 1%, dexamethasone 0.1%, difluprednate 0.05%).
- Topical immunomodulators/Calcineurin inhibitors (cyclosporine 0.05% or 1%, CsA cationic emulsion 0.1%, and tacrolimus 0.03% or 0.1% as ophthalmic ointments or 0.03% or 0.1% as ophthalmic suspension): Significantly effective in controlling chronic inflammation. They act specifically on the Th2 lymphocyte pathway, inhibiting calcineurin, disrupting the activation of the nuclear factor of activated T-cells, and stopping the transcription of IL-2.
- Biological therapies: monoclonal antibodies against IL-4, IL-5, and IL-13 (e.g., dupilumab, mepolizumab, reslizumab, and benralizumab) and IgE (omalizumab) represent a new therapeutic approach in severe cases that are refractory to other therapies.

Regarding systemic therapies:

- Oral antihistaminic (cetirizine, levocetirizine, loratadine, desloratadine). Usually used as adjunctive treatment for mild flare-ups or if the patient also suffers from atopy or allergic rhinitis.
- Oral steroids.
- Oral immunomodulators.

The surgical treatments [61,62,63,64,65]:

- Supratarsal injection of long-acting steroid (triamcinolone acetonide) or surgical excision, use of mitomycin C, cryotherapy of papillae in cases with giant papillae.
- Superficial keratectomy.
- Excimer laser therapeutic keratectomy (PTK).
- Amniotic membrane transplant.
- Limbal stem cell transplant.

In addition to pharmacological treatments, there are a series of non-pharmacological therapies and measures that can help in therapeutic management [60,61]. They include:

- Identify as much as possible and avoid exposure to allergens.
- Compressions with cold water to reduce itching, redness, and swelling.
- Patient education, from how to correctly apply eye drops and other therapeutic agents to maximum eyelid–ocular hygiene.

The aim of treatment is to treat and control acute symptoms, prevent flare-ups, improve the patient’s quality of life, and minimize side effects from drug therapy. Treatment involves lifestyle changes, avoiding potential allergens, and cold compresses. Artificial tears, topical antihistamines, and topical mast cell membrane stabilizers may be used as first-line treatment. In moderate or refractory cases, previous treatments are continued with the addition of short-term, pulsed therapy with topical corticosteroids. In general, steroids should be used in a pulsed manner in acute cases and slowly tapered over 1 to 3 weeks. Once inflammation is controlled, corticosteroids should be tapered and discontinued.

In refractory cases or if steroid dependence or resistance develops, topical immunomodulators such as cyclosporine 0.05% or 0.1% and tacrolimus 0.03% as an ointment or solution may be used. These agents are steroid-sparing and are particularly useful in long-term control. In particularly severe cases, it is possible to use high-potency topical steroids such as prednisolone acetate 1% or dexamethasone, using them for the shortest possible time and under careful surveillance and follow-up for possible side effects, as well as the immunomodulatory agents seen previously. Oral systemic therapy, with antihistamines, steroids, and immunomodulators, may be useful in cases where topical therapy alone is not sufficient to control the disease. This is a step-by-step approach in which the patient is controlled in a relatively short time and the therapy must be modified based on the therapeutic response. To date, there is no univocal therapy even if many exemplifying flow charts are proposed in the literature. In most cases, VKC undergoes spontaneous improvement after puberty; despite this, it is essential to still treat the patient to control the symptoms and pathologies and avoid possible ocular complications [12,60,61,66]. The summary regarding the management of VKC is listed in Table 3.

## 5. Discussion

This systematic review provides a comprehensive overview of VKC, reinforcing that its diagnosis remains fundamentally clinical. According to prior reports, no definitive biomarker has yet been identified for VKC, making patient history and ocular examination the cornerstone of diagnosis. Ancillary allergy testing (e.g., skin prick tests, serum IgE) can help confirm atopy and distinguish VKC from other forms of ocular allergy but these are supportive rather than diagnostic [22]. Our findings echo earlier studies in the literature, that VKC’s classic signs (giant papillae on the tarsal conjunctiva, limbal Trantas dots) and symptoms (intense itching, photophobia, discharge) are highly characteristic. Notably, recent studies highlight the risk of misdiagnosis or delayed diagnosis; for example, severe atopic keratoconjunctivitis can mimic VKC but tends to run a more chronic course. Such insights underscore the need for heightened clinical vigilance, especially in pediatric patients presenting with seasonally exacerbated ocular irritation [67].

Encouragingly, this review also sheds light on emerging diagnostic approaches. Several immunopathological studies have explored tear fluid biomarkers (e.g., eosinophil cationic protein, eotaxin-2, osteopontin, periostin), finding correlations with disease severity. These potential biomarkers, while not yet part of routine practice, expand the diagnostic toolkit and may in the future enable objective disease activity monitoring. Nevertheless, as of now, VKC lacks standardized diagnostic criteria and severity grading scales [60]. Another important finding is the expanding demographic profile of VKC. Historically, VKC has been considered a disease of childhood and early adolescence, often remitting after puberty. Our review supports that paradigm—VKC predominantly affects boys in the first decade of life and commonly quiesces by early adulthood. However, we also identified an increasing number of adult-onset VKC cases, a phenomenon that has been recently described in the literature. These adult patients present with the same clinical features as younger VKC patients, but studies indicate they may experience a more intense inflammatory response and a higher risk of chronic complications (such as conjunctival fibrosis).

Recent studies reported elevated levels of Th1 cytokines (IL-1, IL-2, IFN-γ) and fibrogenic markers in adults with VKC compared to pediatric cases. This observation expands our understanding of VKC’s pathophysiology, suggesting that adult VKC might involve additional immune pathways beyond the classic Th2/allergic profile. Clinicians should therefore be aware that VKC is not strictly pediatric and that late-onset cases may necessitate closer monitoring for aggressive disease course [67,68,69,70]. The therapeutic insights gained from this review affirm the stepwise management approach to VKC while highlighting new advancements in treatment. Mild to moderate VKC is generally managed with topical antihistamines and mast cell stabilizers to control itch and congestion, often supplemented by lubricating agents. These first-line therapies were uniformly recommended across studies and remain comparable to those used in other allergic conjunctivitis. For patients with more severe or persistent diseases, especially those with corneal involvement, short courses of topical corticosteroids are effective in treating acute inflammation. However, our review reinforces the long-recognized concerns about steroid overuse: cataract formation and glaucoma were frequently reported in VKC patients with prolonged steroid therapy.

Earlier guidelines suggest using the lowest effective steroid dose for the shortest duration possible and promptly shifting to steroid-sparing medications. A major focus of recent research, reflected in our findings, is the use of topical immunomodulatory agents as steroid-sparing therapy. Cyclosporine A and tacrolimus in various formulations have become mainstays in the management of chronic VKC, and our review identified numerous studies supporting their efficacy. Both drugs significantly improved ocular surface inflammation and served as effective steroid-sparing treatments, confirming their role as cornerstones of VKC therapy. The introduction of topical tacrolimus for VKC was still experimental in the early 2000s but is now supported by multiple trials and is becoming standard practice, as also reflected in recent international consensus. Beyond traditional agents, our review highlights emerging therapies that could change the VKC treatment landscape. One of the most notable advancements is the exploration of biological medications targeting specific immune pathways. The concept of using biologic agents (like omalizumab) for VKC would have been a foreign concept a decade ago; today, building on the success of biologics in asthma and atopic dermatitis, case reports are translating that success into VKC care.

Omalizumab, an anti-IgE monoclonal antibody originally used for asthma and urticaria, has shown promise in severe VKC. Multiple case series and retrospective analyses reported marked improvements in VKC symptoms and a reduction in steroid requirements in patients treated with omalizumab. In these reports, children with severe VKC experienced a resolution of chronic symptoms and could taper off steroids, with concomitant improvements in quality of life. The benefit is biologically plausible given that about 55–60% of VKC patients have evidence of IgE-mediated hypersensitivity, and omalizumab neutralizes IgE activity. Interestingly, omalizumab may also modulate non-IgE pathways, which is relevant since a substantial subset of VKC patients (around 40–50%) are non-atopic. The collective evidence to date indicates that omalizumab could become a valuable option for refractory VKC that fails conventional therapy. This expands the therapeutic arsenal beyond topical agents and opens the door to systemic, targeted immunotherapy for ocular allergies. Nevertheless, the findings are preliminary. Well-designed clinical trials are needed to confirm the efficacy, optimize dosing, and assess the long-term safety of biologics in VKC.

Clinically, previous studies had suggested that VKC predominantly remits by adolescence, and severe complications were thought to be rare with proper management. Our findings provide nuance to this understanding. We conclude that most children outgrow VKC but we also document that a significant minority continue with active disease into adulthood. Furthermore, while prior publications acknowledged corneal damage (like shield ulcers) as a complication, the true burden of VKC complications and quality-of-life impact has been elucidated more clearly in recent years. The strong association between VKC and keratoconus (with up to one-quarter of VKC patients developing keratoconic changes) has been reinforced in recent studies, emphasizing how chronic eye rubbing and inflammation in VKC can lead to ectasic corneal disease. These comparisons suggest that while the foundational knowledge of VKC has stood the test of time, ongoing research continues to refine our understanding of its full clinical spectrum and long-term consequences.

The synthesis of evidence reinforces the critical importance of early diagnosis and proactive management. Given the risk of sight-threatening complications, even in a small fraction of patients, ophthalmologists should maintain a low threshold to initiate adequate therapy and follow VKC patients closely. The confirmation that no reliable biomarkers exist for VKC means that clinicians must rely on meticulous clinical assessments to gauge disease activity. In practice, this entails regular slit-lamp examinations to monitor corneal involvement (looking for keratitis or ulceration) and conjunctival changes, as well as querying patients (or parents) about symptom control. One immediate application of our findings is the call for implementing structured severity assessments in routine care [71,72,73]. Another impact on practice is the reinforcement of a steroid-sparing mindset in VKC management.

Ophthalmologists are aware of steroid complications, but VKC—with its chronic relapsing course—poses a constant temptation to use long-term topical steroids. The evidence compiled here strongly supports transitioning patients to steroid-sparing agents like cyclosporine A 1% drops or tacrolimus 0.03% ointment as soon as feasible. Many eye care providers have already embraced this strategy but our review provides additional confidence that these agents not only work but are safe for extended use in children, with minimal systemic absorption and only mild local side effects (e.g., transient burning). Moreover, allergists can consider allergen immunotherapy in carefully selected VKC patients—for instance, a dust mite-sensitive child with perennial VKC—as some evidence suggests it can improve outcomes when combined with standard pharmacotherapy (with improvement in symptoms and reduced medication use in 70–80% of patients in one study) [74,75].

Interdisciplinary collaboration is therefore key: ophthalmologists focus on the ocular surface disease, while allergists address systemic allergic inflammation and any novel immunomodulatory treatments like biologics. While this review consolidates a wide range of studies on VKC, it also brings to light several limitations in the current evidence and gaps in our understanding of the disease. One prominent limitation is the heterogeneity of available studies. Many of the included studies differed in their design (ranging from small, randomized trials to case series and expert opinions), patient populations, and outcome measures. This heterogeneity made direct comparisons challenging and sometimes impeded the ability to draw firm quantitative conclusions. Another limitation is that most therapeutic evidence for VKC comes from uncontrolled or short-term studies.

There are relatively few large, double-blind randomized controlled trials (RCTs) in VKC, given the logistical challenges of studying a seasonal, flaring disease that primarily affects children. As a result, many of our treatment recommendations rely on level II or III evidence. The findings of this review point to several important avenues for future research and development in VKC. First and foremost is the establishment of standardized diagnostic and severity criteria. In parallel, research into objective biomarkers for VKC should continue. Advancements in tear film analysis, such as cytokine profiling, proteomics, and microRNA signatures, hold promise for identifying disease activity markers. The development of reliable tears or serum biomarkers could revolutionize VKC monitoring.

There is a scientific imperative to further unravel VKC’s pathophysiology. Genetic studies might identify susceptibility loci or regulatory genes involved in the allergic response of the ocular surface, and other studies are needed to better understand the role of hormones (as suggested by differences in adult VKC), neurogenic factors (the ocular itch cycle), and the microbiome of the conjunctiva. Finally, future research should therefore extend beyond clinical trials and into implementation science: how can we deliver affordable VKC treatment in resource-limited settings? This might include developing low-cost generic formulations of topical immunosuppressants, training primary care providers in early VKC recognition, and creating regional treatment guidelines adapted to local realities.

## 6. Conclusions

In conclusion, this comprehensive review reinforces the primarily clinical nature of VKC diagnosis, emphasizing the continued absence of definitive biomarkers. However, emerging research into tear-based biomarkers and immunological pathways provides promising avenues for objective monitoring and tailored treatments. While VKC predominantly affects pediatric patients and is commonly resolved by adulthood, the identification of adult-onset cases and their distinct immunopathology underscores the complexity of its clinical spectrum. This review highlights the expanding therapeutic landscape, notably steroid-sparing agents such as topical cyclosporine A and tacrolimus, as well as innovative biologic therapies like omalizumab for refractory cases. Despite these advancements, the current evidence remains fragmented and heterogeneous, underscoring the need for standardized diagnostic criteria, robust randomized trials, and equitable access to effective therapies, particularly in resource-limited settings.

## Figures and Tables

**Table 1:** Classification of vernal keratoconjunctivitis.

Grade	Key Symptoms	Principal Clinical Signs	Recommended Management/Follow-Up
**1—Mild**	Mild pruritus; absent-to-slight photophobia; limited tearing	Discrete conjunctival hyperemia; small tarsal papillae; no or minimal corneal epithelial change	Topical antihistamine and/or mast-cell stabilizer; artificial tears; infrequent review
**2—Moderate**	Marked itching; increased photophobia; pronounced foreign-body sensation	Enlarged (“giant”) tarsal papillae; possible limbal—nodules/infiltrates; punctate epithelial erosions or micro-ulcers	Short courses of low-potency topical corticosteroid; add topical immunomodulator (e.g., cyclosporine) if refractory; scheduled review, especially during flares
**3—Severe**	Persistent intense itching; marked photophobia; significant discomfort with QoL impact	Multiple giant papillae; prominent limbal Horner–Trantas dots; recurrent superficial shield ulcers or large infiltrates; eyelid edema, intense hyperemia	Medium-to-high-potency topical corticosteroid for extended periods; continuous topical immunomodulator (cyclosporine or tacrolimus); close follow-up to avert corneal sequelae
**4—Very severe/Refractory**	Debilitating pruritus; extreme photophobia; threat to sustained vision; treatment-related anxiety/frustration	Deep and/or recurrent shield ulcers; dense corneal infiltrates with scarring risk; possible corneal deformation (e.g., keratoconus from rubbing); pronounced conjunctival edema/hyperemia	Intensive anti-inflammatory regimen (high-potency topical steroid ± immunomodulator); systemic immunomodulation if required; very frequent monitoring and multidisciplinary care (ophthalmology + allergy/immune)

**Table 2:** Diagnostic tools used in the management of vernal keratoconjunctivitis.

Diagnostic Tool	Key Features	Advantages	Limitations
**Anterior Segment OCT (AS-OCT)**	- Non-invasive cross-sectional imaging of the cornea and anterior chamber - Detects early corneal thinning, epithelial irregularities, and scarring	- Well tolerated by most patients - Rapid scan acquisition - Widely available in tertiary centers	- High initial cost - Requires expertise in image interpretation - Sedation may be needed in very young children
**In Vivo Confocal Microscopy (IVCM)**	- Provides cellular-level images of corneal and conjunctival structures - Detects inflammatory cell infiltrates and nerve alterations	- High-resolution details of the ocular surface - Enables tracking of response to therapy over time	- Operator-dependent technique - It may be uncomfortable for children - Requires costly, specialized equipment
**Corneal Topography**	- Identifies subtle corneal curvature changes indicative of keratoconus - Visualizes localized corneal thinning	- Useful for early detection and monitoring of ectatic changes - Guides timely interventions (e.g., corneal crosslinking)	- Equipment can be expensive - Requires patient cooperation - Multiple measurements over time needed for best accuracy
**Tear Biomarker Assays**	- Evaluates cytokines, chemokines, and immunoglobulins in tear samples - Can reveal patient-specific inflammatory profiles	- Potentially allows for personalized therapy decisions - May help identify flare risk and disease severity	- Not widely available outside specialized centers - Sample collection in pediatric patients can be difficult - Requires advanced laboratory facilities

**Table 3:** The management of vernal keratoconjunctivitis.

Phase	Description
**1. Clinical Evaluation**	- Medical history - Slit-lamp examination - Symptoms
**2. Advanced Diagnostic Imaging**	- Tear film analysis - Anterior segment OCT (AS-OCT) - In vivo confocal microscopy (IVCM) - Corneal topography
**3. Laboratory Tests and Biomarkers**	- Cytokine measurement - Lacrimal IgE - Conjunctival cytology/impression cytology - Presence of eosinophils - Goblet cell density
**4. Disease Grading and Risk Stratification**	- Clinical scoring systems (e.g., Zicari) - Multimodal imaging
**5. Personalized Treatment Plan**	- First-line treatment: topical steroids, mast cell stabilizers, topical antihistamines. - In moderate-severe cases: immunomodulatory therapy - For refractory cases: topical cyclosporine, tacrolimus, biologics (anti-IgE, e.g., omalizumab)

## Data Availability

No new data were created or analyzed in this study. Data sharing is not applicable to this article.
